# Disruption in Thyroid Signaling Pathway: A Mechanism for the Effect of Endocrine-Disrupting Chemicals on Child Neurodevelopment

**DOI:** 10.3389/fendo.2018.00204

**Published:** 2018-04-30

**Authors:** Akhgar Ghassabian, Leonardo Trasande

**Affiliations:** ^1^Department of Pediatrics, New York University School of Medicine, New York, NY, United States; ^2^Department of Environmental Medicine, New York University School of Medicine, New York, NY, United States; ^3^Department of Population Health, New York University School of Medicine, New York, NY, United States; ^4^NYU Wagner School of Public Service, New York, NY, United States; ^5^NYU College of Global Public Health, New York University, New York, NY, United States

**Keywords:** thyroid, endocrine disrupting chemicals, neurodevelopment, children, brain

## Abstract

Thyroid hormones are crucial in normal brain development. Transient and mild thyroid hormone insufficiency in pregnancy is also associated with impaired neurodevelopment in the offspring (e.g., 3–4 IQ score loss in association with maternal free thyroxine in the lowest fifth percentile). While inadequate iodine intake remains the most common underlying cause of mild thyroid hormone insufficiency in vulnerable populations including pregnant women, other factors such as exposure to environmental contaminants have recently attracted increasing attention, in particular in interaction with iodine deficiency. Endocrine-disrupting chemicals (EDCs) are natural and synthetic substances with ubiquitous exposure in children and adults including pregnant women. EDCs interfere, temporarily or permanently, with hormonal signaling pathways in the endocrine system by binding to hormone receptors and modifying gene expression. Other mechanisms involve alterations in production, metabolism, and transfer of hormones. Experimental studies have shown that exposures to EDCs affect various brain processes such as neurogenesis, neural differentiation and migration, as well as neural connectivity. Neuroimaging studies confirm brain morphological abnormalities (e.g., cortical thinning) consistent with neurodevelopmental impairments as a result of EDC exposures at standard use levels. In this review, we provide an overview of present findings from toxicological and human studies on the anti-thyroid effect of EDCs with a specific attention to fetal and early childhood exposure. This brief overview highlights the need for additional multidisciplinary studies with a focus on thyroid disruption as an underlying mechanism for developmental neurotoxicity of EDC, which can provide insight into modifiable risk factors of developmental delays in children.

Observations of children born with cretinism in iodine deficient areas prompted scientists to discuss the impact of early thyroid function on brain development (1). Decades later, evidence confirmed that undetected or inadequately treated thyroid deficiency in pregnant women was associated with impaired cognition in the offspring, even in the absence of neonatal hypothyroidism (2). A series of influential studies by Morreale de Escobar and colleagues using experimental animal models identified structural and functional abnormalities in the cerebral cortex and the hippocampus due to low thyroid hormones during gestation (3–5). Recently, epidemiological studies confirmed that transient as well as mild thyroid hormone insufficiency during critical windows of brain development were also associated with impaired cognition, psychomotor and language development, behavioral problems, and abnormal cortical and subcortical morphology (6–12).

While inadequate iodine intake remains the most common cause of thyroid insufficiency worldwide (13), other factors including autoimmunity (14) and environmental chemicals in routine doses of exposure have recently attracted increasing attention (15, 16). Several experimental studies have shown that exposure to endocrine-disrupting chemicals (EDCs) affects neural differentiation and migration and neural connectivity (17). Growing evidence on adverse health effects of certain EDCs, such as polychlorinated biphenyls (PCBs) and polybrominated diphenyl ethers (PBDEs), has led to stringent policies to control exposure. EDCs interfere with thyroid function at different levels including the central regulatory system in the hypothalamus and pituitary, thyroid hormone production at the thyroid gland, thyroid hormone transfer, as well as hormone bioavailability, function, and metabolism (16) (Figure 1, Table 1). Many EDCs pass through the placenta and blood–brain barrier and are also secreted in breastmilk (18, 19). Since circulation of thyroid hormones in the cerebral spinal fluid (CSF) resembles of the levels in the serum, EDCs potentially interfere with thyroid hormone carriers in the CSF when cross the blood brain barrier. Here, we briefly review seven groups of substances with anti-thyroid activities.

**Figure 1:**
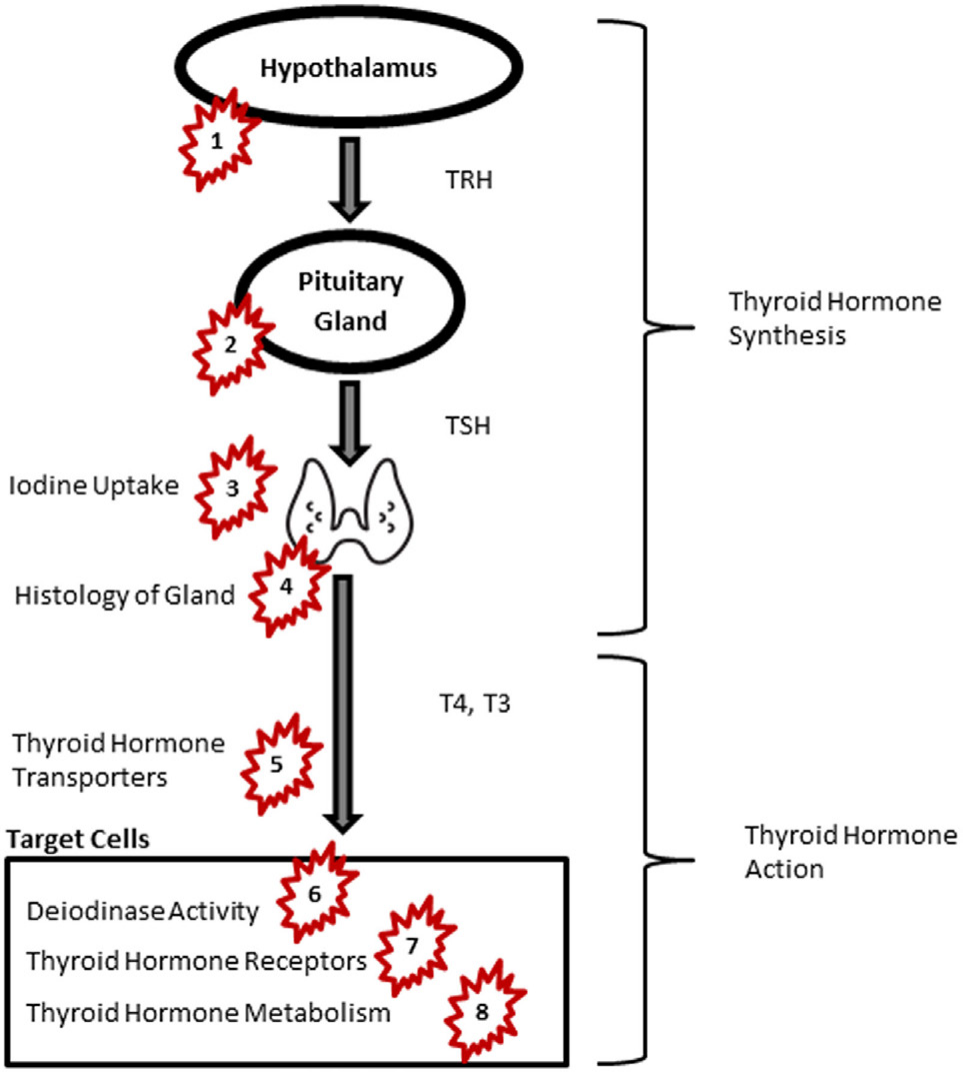
Thyroid signaling pathway and endocrine-disrupting chemicals. Groups of chemicals act at: PCB and PCDD: 5, 7; PBDEs: 5, 6, 7, 8; pesticides: 4, 5, 7; PFASs: 5, 6; NIS: 3; BPA: 2, 7; phthalates: 1, 2, 5, 8. Abbreviations: BPA, bisphenol A; NIS, sodium iodide symporters; PBDE, polybrominated diphenyl ethers; PCB, polychlorinated biphenyl; PCDD, polychlorinated dibenzodioxins; PFAS, perfluoroalkyl substances; TRH, thyroid-releasing hormone; TSH, thyroid-stimulating hormone; T4, thyroxine; T3, triiodothyronine. Image of thyroid: by P. J. Witt, AU from the Noun Project, Creative Commons.

**Table 1:** Endocrine disrupting chemicals (EDCs) and target of action in the hypothalamus–pituitary–thyroid axis.

Groups of EDCs	Target of action
Polychlorinated biphenyls and polychlorinated dibenzodioxins (PCDD)	Thyroid hormone transportation Thyroid hormone receptors

Polybrominated diphenyl ethers	Thyroid hormone transporters Deiodinase activity in the thyroid gland Thyroid hormone receptors Thyroid hormone metabolism

Pesticides	Histology of thyroid gland Thyroid hormone transportation Thyroid hormone receptors

Perfluoroalkyl substances (PFASs)	Thyroid hormone transportation Deiodinase activity in the thyroid gland

Sodium iodide symporters (NIS)	Iodine uptake into the thyroid gland

Bisphenol A and other phenols	Expression of thyroid receptor genes in the pituitary Thyroid hormone receptors

Phthalates	Thyroid-releasing hormone receptor in the hypothalamus and pituitary Thyroid-stimulating hormone receptor in the thyroid gland Expression of genes related to thyroid hormone metabolism, synthesis, and transportation

## PCB and Polychlorinated Dibenzodioxins (PCDD)

Polychlorinated biphenyls and polychlorinated dibenzodioxins (PCDD) are persistent organic pollutants, which are no longer produced due to their carcinogenic effects (20), but are present in several products manufactured prior to banning (21). PCBs and their metabolites bind to thyroid transport proteins such as transthyretin and interfere with thyroid hormone transport (22). The action of PCBs at the level of thyroid receptor (TR) activity is both agonistic and antagonistic, depending on PCB congeners and the target tissue. Specific PCB metabolites, if hydroxylated by the metabolic enzyme cytochrome P450 1A1, act as TR agonists and impact mRNA expression of well-known thyroid hormone-response genes in the liver and in the pituitary (23). PCBs can also bind to TR and antagonize triiodothyronine (T3) inhibiting TR-mediated gene activation. Experimental studies in rats have shown that thyroid regulated events during early development, e.g., neuronal migration in the cortex, are disturbed with PCB exposure (24). PCBs, in concentrations commonly observed in humans, interfere with thyroid hormone receptor signaling (mainly through TRβ complex) and disturb oligodendrocyte differentiation and white matter maturation during early development (25).

Substantial evidence from epidemiological studies has shown associations between PCB or PCDD exposures and abnormal thyroid parameters. Cross-sectional examination of serum PCB and dioxin levels and thyroid parameters have shown positive associations between serum thyroid-stimulating hormone (TSH) and PCB congeners and PCDD and negative associations between PCBs and T3 and thyroxine (T4), with stronger associations among women (26–29). In cord blood, there is an inverse association between concentrations of organochlorine compounds and free T3 (fT3) and free T4 (fT4), but not TSH (30). Similar associations are shown in longitudinal studies (31, 32). Positive associations between serum PCB and T3 and T4 (33–35) and null associations between serum PCB and thyroid parameters are also reported (36). Methodological considerations (e.g., standardizing methods) might explain the differences in the observed associations (37). Overall, human studies support findings from animal models and show that concentrations of PCB and PCDD at levels commonly detected in the general population can interfere with thyroid function. Further studies are still needed, however, to assess whether the observed effects of PCBs on early brain development (38, 39) are partly (thyroid disruption together with direct neurotoxicity or other mechanism) or fully (entirely through disruption in thyroid signaling pathway) explained by their thyroid disrupting activities.

## Polybrominated Diphenyl Ethers

Polybrominated diphenyl ethers are organic compounds that are used as flame retardants in building materials, electronics, furnishing, and textiles. The usage of some PBDE congeners is increasingly being controlled; however, due to their high resistance to degradation processes, people are still exposed to PBDEs. PBDEs have a similar chemical structure to thyroid hormones. Experimental studies have shown that PBDEs, at environmentally relevant doses, bind to receptors, and inhibit binding of T3 to TRs, and suppress T3 actions (40, 41). Other mechanisms of actions are a competitive binding for serum transporters (e.g., transthyretin and thyroid binding globulin), upregulation of clearance enzymes (e.g., glucuronidases) and liver metabolism, and inhibition of thyroid deiodinase activity (42, 43). In zebrafish, decabromodiphenyl ether (BDE-209) exposure in parents induces a decrease in T4 and a downregulation in expression of TR genes (44). Similar inverse associations between exposure to PBDE congeners and thyroid hormones have been reported in other studies (45–47). While human studies confirm thyroid disruption by PBDEs, the direction of this association varies across studies and PBDE congeners. For example, BDE-153 levels have been inversely associated with first trimester total T3 (29) and TSH in pregnant women [but not with fT4 and total T4 (TT4)] (48). Another longitudinal study has shown positive correlations between maternal BDE-47, BDE-99, and BDE-100 during pregnancy and T3 in cord blood (39). Both higher and lower levels of T3 have been reported in association with PBDE exposure in pregnant women (49, 50). A decrease in cord blood fT4 and maternal TT4 and fT3 at delivery are shown in relation to maternal PBDE exposure in early pregnancy (51). Considering that several epidemiological studies have shown the impact of prenatal PBDE exposure on neuropsychological, motor, and cognitive functioning in children (39, 52, 53), thyroid disruptive properties of PBDE congeners can be one of the underlying mechanisms for the adverse effect on early brain development.

## Pesticides

Individuals are widely exposed to pesticides. Several pesticides are currently banned; however, they are still detectible in the environment from previous use (54). Chlorpyrifos, the top selling pesticide in the United States (US) have been increasingly used for corn production despite stringent regulations for domestic use (55). Toxicological evidence has shown long-term disruption of thyroid function by these chemicals (56–58) and human studies have confirmed the association (59, 60). The potential actions on the thyroid system are the perturbation of thyroid hormone transport as well as histological and histomorphometrical effects on the thyroid gland (58, 61). There is strong epidemiological evidence that show the effect of prenatal exposure to chlorpyrifos and other pesticides on neurodevelopment, including childhood tremor, delayed psychomotor and mental development, IQ loss, and ASD (62–65). A neuroimaging study in 40 six- to eleven-year-old children has found that higher neonatal levels of chlorpyrifos are associated with several brain morphological abnormalities including cortical thinning and abnormal morphological measures of cerebral surface, consistent with neurocognitive findings (66). Mechanistic studies and comparison of chlorpyrifos exposure neuroimaging findings with brain influences of thyroid disruption will further unravel the mechanism for the neurotoxicity of chlorpyrifos and other pesticides.

## Perfluoroalkyl Substances (PFASs)

Perfluoroalkyl substances are persistent chemicals which are widely used in textiles, furniture, and cookware (67, 68). Since 2002, major US companies have been phasing out two PFASs, perfluorooctanoic acid (PFOA), and perfluorooctane sulfonate (PFOS). Nonetheless, PFOS and PFOA were detectible in children from the National Health and Nutrition Examination Survey (NHANES) 2013–2014 (69) suggesting that children born after voluntarily discontinuation have been exposed to PFASs. PFASs interfere with bindings of thyroid hormone to transthyretin (70) and upregulate deiodinase in the thyroid gland (71). Serum PFOS and PFNA have been associated with an increase in TT4 (72). Cord blood perfluoro *n*-pentanoic acid has been positively associated with cord blood TT4 (73). In a sample of newborns, girls in the highest quartile of PFOA exposure during prenatal period showed increased T4 levels compared to the lowest quartile (74). In another study, cord blood concentration of perfluoro *n*-pentanoic acid and perfluorohexane sulfonic acid was associated with increased T4 and T3 levels (in the cord blood), while PFNA was associated with decreased TSH concentration in newborn girls (73). Inconsistent results have been reported regarding the effect of PFASs on child neurodevelopment such as cognition, behaviors and executive function, developmental milestones, psychomotor development, and academic achievement (75). While some studies showing no association between maternal serum PFOA and PFOS in early pregnancy and child neurodevelopment (76–79), others found positive associations (80–83). Quaak et al. reported a sex-specific effect (84). Thyroid disruption together with influences on other parts of the endocrine system might explain these sex-specific effects (85, 86).

## Sodium Iodide Symporters (NIS)

Perchlorate, thiocyanate, and nitrate competitively inhibit the NIS—a transmembrane protein responsible for iodide uptake into the thyroid gland at the membrane of thyroid follicular cells. They interfere with thyroid iodine uptake, and affect thyroid hormone production and bioavailability (87). Individuals are exposed to these contaminants through food or other sources (e.g., cigarette smoke for thiocyanate or rocket propellant and fertilizers for perchlorate and nitrate). Though these chemicals do arise naturally, anthropogenic activities are a major source of exposure. In the 2001–2002 NHANES data, there was an inverse association between urinary levels of perchlorate and TT4 and a positive association with TSH (88). A similar positive association has been reported between urinary perchlorate, nitrate, and thiocyanate and fT4, with indications for a sex-specific effect (89). Adolescent boys and girls are vulnerable subpopulations to thyroid-blocking effects of NIS inhibitors (90). In the Controlled Antenatal Thyroid Screening Study, maternal perchlorate in the first trimester of pregnancy have been associated with reduced IQ in the children of hypothyroid or hypothyroxinemic pregnant women, suggesting that high exposures during sensitive windows of brain development in combination with maternal thyroid status might adversely influence neurodevelopment (91).

## Bisphenol a (BPA) and Other Phenols

Bisphenols are organic synthetic compounds widely used in the production of aluminum cans, plastics, thermal paper receipts, and food packaging. Bisphenol A is detectible in >90% of urine samples in the US population (92). BPA is a weak estrogenic substance (93) but also interferes with thyroid function (94, 95). *In vivo*, BPA can impair thyroid hormone action by antagonizing T_3_-induced TR activation (TRα1 and TRβ1) and by suppressing its transcriptional activity in a dose-dependent manner (94). Another study suggests that BPA acts selectively as TR antagonists on TRβ—independent of its estrogenic effects—causing serum T_4_ to rise (95). Prenatal exposure to BPA in rats is shown to upregulate TRα mRNA expression in the fetal forebrain and alters neuronal migration patterns during corticogenesis (96). BPA and its structural analogs cause dysregulation of TR gene expression in pituitary cells and thyroid gland (97). Triclosan affects thyroid hormone-dependent metamorphosis in animals (98).

Cross-sectional studies have shown that higher urinary levels of BPA are associated with lower fT4 and TSH (99–102). In a cohort of pregnant women, modest associations have been reported between higher BPA and lower TT4, if measurements were close in time (103). In follow-up studies, negative correlations have been shown between maternal BPA and TSH in newborns (104). In boys, an inverse association has been shown between maternal BPA in the third trimester and serum TSH (103). The latter is in line with findings of epidemiological studies that have shown the sex-specific association between prenatal BPA and child behavior (105–110). In sum, BPA dysregulates thyroid function leading to a positive association with T3 and an inverse association with T4 and TSH. Estrogenic effects or other sex hormone disruption mechanisms of BPA exposure on the offspring’s thyroid parameters might explain differential effects observed on behavioral outcomes of boys and girls.

## Phthalates

People are ubiquitously exposed to phthalates, non-persistent synthetic chemicals that are used in plastic and consumer products such as cosmetics, adhesives, and detergents. Some forms of phthalates such as di-2-ethylhexylphthalate (DEHP) are no longer used in baby toys; yet phthalates metabolites remain detectible in individuals across age groups (111, 112). Urinary phthalates are cross-sectionally associated with lower fT4 and higher TSH (99, 113, 114). One study has found an inverse association between non-DEHP and fT4 in girls only (113) and another study has reported inverse associations between DEHP concentrations and fT4 in girls (115). In the later, urinary concentrations of dibutyl phthalate have been inversely associated with fT3 in boys (115). Higher concentrations of phthalates in maternal prenatal urine samples have been associated with lower thyroid hormones (116). Longitudinal studies in pregnant women have also found that phthalate metabolites are inversely associated with TSH (117, 118) and positively associated with fT4 and TT4 (118). Null associations between prenatal DEHP exposure and infant thyroid hormones have also been reported (119).

Despite data suggesting thyroid dysregulation resulting from phthalate exposure, specific underlying mechanisms are poorly understood. A recent experimental study in rats has shown that DEHP can downregulate the thyroid-releasing hormone (TRH) receptor in the hypothalamus, upregulate the protein and mRNA levels of TRH receptor in the pituitary, and downregulate mRNA expression of TSH receptors in the thyroid (120). In zebra fish larvae, phthalate exposure alters the transcription of genes in the hypothalamic–pituitary–thyroid axis resulting in an increased T3 and decreased T4 (121). To confirm the extent to which these findings from animal models translate into humans further studies are needed. Considering the potential link between phthalates and impaired neurodevelopment (122–125), future studies should focus on thyroid dysfunction as a mediating factor.

Concern is growing regarding the long-lasting effect of chemicals, routinely found in the environment, on the fetal and child brain through anti-thyroid capacities. Thyroid disruption is of particular interest because several EDCs interfere with thyroid function in a sex-specific manner, which might explain the sexual dimorphism in the brain effect of EDCs. EDCs comprise of various compounds with different mechanisms of anti-thyroid effects. This might explain a heterogeneous neurodevelopmental outcomes associated with EDCs. Thyroid disruptive effects of chemicals in combination with or independent of iodine deficiency is another topic which has been sparsely considered in epidemiological settings.

Additional multidisciplinary studies with a focus on thyroid disruption as an underlying mechanism can strengthen the existing knowledge on the neurotoxicity EDC. Two such interdisciplinary efforts have been started in Europe and in the US. The LifeCycle Project is a European network of population-based birth cohorts which aims to examine the impact of early life stressors on health and development using a life course approach. In the US, the Environmental Influences on Child Health Outcomes (ECHO) program uses information from 50,000 children and their families from across the US “to enhance the health of children for generations to come” (126). This information comprises several key elements including demographics, environmental exposures, biological measures, and child health outcomes. ECHO, the LifeCycle Project and similar efforts can be used as a valuable platform for mechanistic studies of EDC exposure and child neurodevelopment, which subsequently provide insight into modifiable risk factors of developmental delays in children. Such research will further clarify the unfavorable effects of EDCs in the context of dietary factors and other health conditions such as autoimmunity (127, 128).

## Author Contributions

AG and LT drafted the manuscript and reviewed the final draft for submission.

## Conflict of Interest Statement

The authors declare that the research was conducted in the absence of any commercial or financial relationships that could be construed as a potential conflict of interest. The handling Editor and reviewer BD declared their involvement as co-editors in the Research Topic, and confirm the absence of any other collaboration.
